# Supplementary material for: Sonochemical Synthesis of Cu@Pt Bimetallic Nanoparticles
Source: Molecules. 2022 Aug 18;27(16):5281. doi: 10.3390/molecules27165281 (PMC9415347; doi:10.3390/molecules27165281)
Supplement: Supplementary file 1 [file molecules-27-05281-s001.zip › molecules-1820894-supplementary.pdf]

Supporting Information

Cyclic voltammogram acquired at 400 mV s^{-1} for sonochemically synthesized carbon supported Cu-nanoparticles after the addition of $PtCl_4$ is shown in Figure S1. The characteristic Pt-behaviour confirms the presence of Pt in the Cu@Pt-sample.

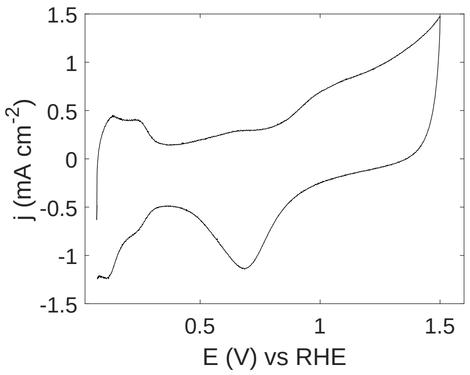

Figure S1. Cyclic voltammogram for sonochemically synthesized carbon supported Cu-nanoparticles after the addition of $PtCl_4$. Voltammograms were acquired in 0.5 mol dm^{-3} H_2SO_4 with a scan rate of 400 mV s^{-1}.

Absorbance spectra showing the development of the Ti-complex formed when $TiOSO_4$ reacts with sonochemically generated H_2O_2 are shown in Figure S2. The constant increase in absorbance suggests a constant generation of ·OH, which in turn form hydrogen peroxide in a pure water solution.

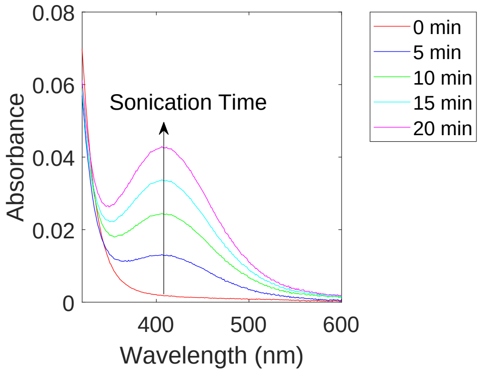

Figure S2. Absorbance spectra of the Ti complex formed when $TiOSO_4$ reacts with H_2O_2. Spectra were acquired for different sonication times.
